# The “Letheon” at the New York Hospital

**Published:** 1847-01

**Authors:** 


*The "Letheon" at the New York Hospital.*—The inhalation of the ætherial compound recently patented by Morton, a dentist of Boston, for the purpose of inducing insensibility to pain during the performance of surgical operations, was tried to-day on a female, from whom two teeth were extracted. A dentist of this city, who has been appointed agent for the above patent—the inhaler and its contents—was present on the occasion. After some explanatory remarks relative to its peculiar properties, mode of administration, etc., he proceeded to exhibit its effects.

The inhaler consists of a glass globe, six or eight inches in diameter; it has two valves, one of which is attached to the mouth-piece and the other to the opposite side. Within this vessel is a large piece of sponge, and an ounce or two of liquid which has the odour of æther. The mouth piece having been placed within the lips of the patient, she was directed to breathe naturally, the nostrils being closed. During the inhalation, she coughed slightly once or twice, and required some persuasion to induce her to continue the process sufficiently long to produce the desired effects. After the lapse of six or eight minutes, she appeared to be in a state resembling that of intoxication; at this moment she was directed to open her mouth, and the teeth were rapidly extracted. The manifestations of apparent suffering, differed in no respect from what is usually seen during similar operations, though on being interrogated when consciousness had partly returned, she acknowledged that she had felt little or no pain; that she knew nothing more of what had been done to her, after she was told to open her mouth.

On being questioned as to the state of her feelings, whilst under the influence of the vapour, whether they were pleasant or otherwise, she remarked that they were very unpleasant; this, the operator stated, was altogether different from what is usually experienced, the sensations being of the most delightful kind. When she arose to leave the theatre, she staggered like a person under the influence of liquor, and the assistance of two individuals was necessary to enable her to proceed.

The result of this trial, seems to confirm what has been stated in reference to the anodyne properties of this ætherial vapour, and that to a certain extent it renders the patient almost wholly insensible to pain. Though such be the fact, it cannot be denied that the employment of this *patented* nostrum by the profession—we mean those of it, who, by the position or rank they hold, are entitled to more than ordinary consideration and respect—is a wide departure from propriety, and the strict observance of the code of medical ethics. What now, we ask, is to prevent any member of the profession from using in his practice, any one, or all, if he pleases, of the patented medicines which he finds daily advertised and puffed in the newspapers, especially after the example set him by several of the surgeons of the two largest hospitals in the country? These gentlemen, have, we fear, incautiously ventured upon slippery ground, and it would be well for them to pause and reflect ere they proceed further."

[We have given place to the preceding statement, kindly furnished us by an eye witness, because we thought, that as public chroniclers of passing events, we were bound to furnish our readers with a notice of what was doing in a matter now exciting general interest. The results appear to be confirmatory of those obtained elsewhere by the use of the Letheon, although the gentleman under whose auspices they were undertaken, has been heard to express himself as by no means satisfied, that it would be found equal to what is anticipated by more sanguine experimenters. It is not our intention to make any comments, since no public testimony of approbation will probably follow. Moreover, the Letheon has as yet been confined to operations in dentistry. We do not object to dentists making use of this vapour, but referring to our preceding remarks upon the subject, we cannot but rejoice, that an accident has deprived us of the necessity of applying to a surgeon of the New-York Hospital, the remarks we felt bound to make on the conduct of those of a similar institution in a neighboring city. It is true that the fluid has since been used, in the case of a patient upon whom a camphor moxa was burned, and with its customary tranquillizing effect, and that this trenches a little more closely upon the domains of our own science. But even this is a small matter, and as we flatter ourselves that no further trials will be made, so long as the secret is kept, we shall dismiss it *sub silentio*, and willingly leave the Letheon, so far as the New-York Hospital is concerned, to the operation of those Lethean waters which will soon, no doubt, flow into it, and which, mingling with it, as with those of a whilom celebrated watery predecessor, will speedily so dilute it, as to lessen at once its dangers and its supposed efficacy.

Meanwhile, as was to be expected, the novelty has been eagerly caught at, as novelties ever are, and sundry persons have been in haste to make hay while the sun shines. Everything now a-days, must be introduced to the public, as well as to the profession, and Dr. Kimball, the agent for the Letheon in this city, has edified the world in a long article in a city paper, upon its virtues. It appears from this, that two surgeons of this city, one of them placed upon the very highest pinnacle of surgical fame, have successfully used the Letheon in their private practice. We can only regret that these gentlemen did not take the same view of employing patented and secret nostra as ourselves, a view, which, it gratifies us much to find, meets with very general favour in the minds of the profession.

It is pleasing to the philanthropist, who must appreciate Dr. Kimball's disinterested zeal in the matter, to be told that Dr. K. is constantly employing it at his house, No. 522 Broadway, with the most *gratifying results.* But perhaps the most remarkable recent instance of the application of this celebrated sedative, is that in which it was administered to a patient, who underwent, under its influence, the removal of a cancerous mamma. The case will doubtless be submitted hereafter to the medical profession, through an appropriate medium; but the philanthropic haste of several editors of newspapers, who had been "invited" to be present, has forestalled the operator, Dr. C. T. Collins, of this city, and a circumstantial account has already appeared in several of the daily papers. The patient, we are told, was insensible to the whole operation, and with the editor of the *Sun*, we "congratulate the accomplished and humane Dr. Collins," on his success. These congratulations are mingled, however, with a dread, that among certain prejudiced persons in the profession, who cling with tenacity to obsolete and antiquated notions of propriety, there may be found some fault, that editors of daily secular papers should have been invited to witness a surgical operation, without being cautioned that they were not to suffer any account of it to appear in the prints under their control, a thing not only unusual, but liable to the suspicion of having been done for the sake of giving éclat to the operator, rather than to the Letheon; as tending slightly towards charlatanry; opposed to that nice sense of rectitude of conduct possessed by the persons aforesaid, and detracting in some degree from that sole regard to the interest of the patient, which it is thought by them should characterize the actions of every high-minded and honourable medical man. Such persons might be disposed (which we are not) to attribute to Dr. C. himself, some hand in this matter, and to opine that such conduct ill became one who had been himself a teacher of others, and the pages of whose journal teem with well directed attacks against the quackeries of the day. Speaking of Homœopathy, (p. 363 of the *Reporter*,) Dr. Collins expresses his belief, that "it would have fallen to the ground long since, had it not been for those of the regular profession, who previously had little to do, and possessed but little, if any real honesty, and took up the practice because it was *something new*, and that money was the *ne plus ultra* of their ambition." Now, there may be, we repeat, persons in the profession, who might be disposed to connect remarks of this kind, with the case in question, and therefore, acquitting, as we do most cheerfully, Dr. Collins of all participation in this extra professional publicity, and attributing it wholly to the intemperate haste with which the editors of our smaller prints seek to communicate information to their readers, or to be more charitable, to the delight felt by them as philanthropists, in the pain-saving process which they had just witnessed, we feel some regret that they were not cautioned against making the publications which they have done. It is perhaps proper to say, that in the little heading to the note subsequently published by Dr. C., the editor of the *Sun* states, that he attended the operation, "at the invitation of a friend."

We had hoped in this number of the Annalist, to have noticed the papers of Drs. Warren and Bigelow, in the No. of the *Bost. Med. Journ.* for December 9th, but space fails us. In our next we will consider them, and with some general remarks on the subject of medical patents, take leave of the unpleasant topic altogether. The following paragraph we append by authority :]

The newspapers of the city have published accounts of some experiments in the New York Hospital, in the case of a new nostrum for rendering patients insensible during surgical operations. We learn that this experiment was made without any consultation of the Surgeons of the Hospital, or any previously expressed concurrence on their part.—*New York Annalist.*

